# Heterogeneity of pneumococcal phase variants in invasive human infections

**DOI:** 10.1186/1471-2180-6-67

**Published:** 2006-07-26

**Authors:** I Serrano, J Melo-Cristino, M Ramirez

**Affiliations:** 1 Instituto de Microbiologia, Instituto de Medicina Molecular, Faculdade de Medicina de Lisboa, Lisboa, Portugal

## Abstract

**Background:**

*Streptococcus pneumoniae *can be carried asymptomatically in the nasopharynx of its human host but can also cause a wide range of infections. A role for pneumococcal phase variants in the different lifestyles of this bacterium has been suggested but no systematic survey of the colony phenotypes of isolates associated with human infections has been undertaken.

**Results:**

We report the colony opacity phenotypes of a genetically diverse set of 304 invasive isolates representing 10 serotypes. Over half of the isolates (52%) presented the opaque phenotype whereas transparent variants accounted for only 26% of the total. However, the frequency of recovery of each phase variant was not uniform, while serotypes 1, 4, 12B and 23F presented the opaque phenotype more frequently than expected by chance, serotypes 3 and 14 where less frequently associated with this phenotype.

**Conclusion:**

The opaque phenotype was the most frequent phenotype found among invasive isolates. An unexpected and equally important finding is the variability of the dominant opacity phenotype found among serotypes. This observation highlights the heterogeneity of opacity phenotypes in invasive isolates and lends further support to the proposal that other factors, in addition to the site of isolation, determine the opacity phenotype of a given isolate. The association between serotype and colonial opacity could help explain epidemiological differences observed among pneumococcal serotypes such as a higher invasive disease potential.

## Background

The only habitat identified for *Streptococcus pneumoniae *(pneumococcus) is the human host where the bacterium is able to persist and multiply, either asymptomatically in the mucosal surface of the nasopharynx or being responsible for infections that range from milder manifestations such as otitis media to life threatening infections such as bacteremia. The transition between the mucosal surface and the blood, two very different habitats, is thought to lead to changes in the physiology of the bacterium and its surface components. Recently, a phenomenon of intrastrain phase variation in the colonial opacity of the pneumococcus has been identified and related to increased survival in each of these habitats [1]. Studies on animal models revealed that the transparent variants (T) persist in the nasopharynx in vivo and show greater adherence in vitro to human pharyngeal and lung epithelial cells [2,3]. On the other hand, experiments performed with an adult mouse model of sepsis showed a strong selection for organisms with the opaque morphology (O) during invasive infections [2]. A recent report however proposes a more complex role for phase variation in the biology of pneumococcus [4]. The authors suggest that the recovery of mainly transparent pneumococci from the nasal cavities of colonized mice may be due to the fact that these could be more easily detached than the opaque variants. In fact it is proposed that O variants play an important role in colonization of the mouse mucosa and that the presence of these variants may be essential for long-term colonization.

The difference in appearance of bacterial colonies is assumed to result from the spontaneous and reversible phase variation of surface components that are selected at different stages of infection and although the genetic basis for this variation are unknown it is clear that the frequency of switching is highly variable from isolate to isolate, ranging from 10^-3 ^to 10^-6 ^per generation [1].

The increased adherence of the T variants was attributed to decreased levels of capsular polysaccharide and increased amounts of cell-surface adhesins [2] whereas O variants have increased levels of capsular polysaccharide that correlate with increased resistance to opsonophagocytosis and ability to cause sepsis [5]. Both DNA microarray technologies and protein two dimensional gel electrophoresis have identified a number of additional differences between the two phase variants whose impact in the two phenotypes remains to be defined [3,6]. Two recent papers document further differences between phase variants of isogenic strains with an increased expression of a neuraminidase (NanA) being associated with the T phenotype [3] and a higher proportion of unsaturated lipids being found in the membrane of the O phenotype [7].

In spite of the abundant information concerning the different expression patterns of each of the phase variants, these were obtained using a small set of strains and only a single study has so far attempted to determine the opacity phenotype of pneumococcal isolates associated with carriage and infection of the human host [8]. In this first study of 19 paired isolates an association of the T phenotype with colonization of the nasopharynx was apparent, however the overwhelming majority of the patients were either HIV positive or had an HIV status unknown (n = 17/19) casting doubts on the relevance of these findings in immunocompetent hosts [8]. Moreover, the isolates expressed mainly serotypes 1 and 4 (n = 13/19) of the 90 different serotypes known to be found in the pneumococcus leaving open the possibility that other serotype specific properties influenced the results.

In this report we examine the prediction from the animal model experiments that strains recovered from invasive infections in humans will be found to be predominantly of the O phenotype using a recent collection of invasive isolates (recovered from normally sterile sites). Our results confirm the animal model predictions but also show heterogeneity in the frequencies of recovery of each phase variant between serotypes, raising the possibility that some serotypes express preferentially one of the phase variants.

## Results

### Colony morphology and relationship with serotype

**Figure 1:**
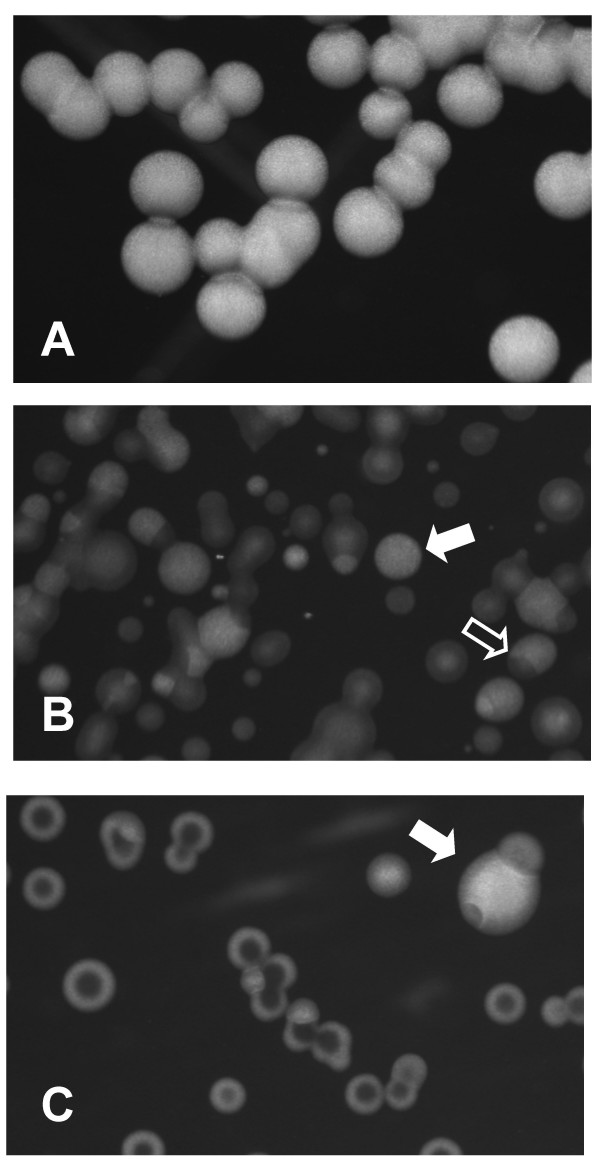
**Pneumococcal colonies exhibiting the various opacity phenotypes**. A) Serotype 12B displaying the opaque phenotype. B) Serotype 14 displaying a mixture of opacity phenotypes. Most colonies are transparent, white arrow indicates an opaque colony, open arrow indicates a sectored colony. C) Serotype 8 displaying mostly transparent colonies. White arrow indicates an opaque sectored colony.

Isolates representing the serotypes tested and the phenotypes found are shown in figure 1 and in additional file 1. Different serotypes displayed noticeable variations in the presentation of the various colony opacity phenotypes in agreement with previous observations [1,8]. Nevertheless, in spite of this variability the presence of multiple colony morphology variants in the same set of plates allowed unequivocal identification of the dominant phenotype of each isolate. The dominant colony morphology of the isolates analyzed is summarized in the table. Over half of the isolates (52%, n = 158) were classified as O variants and only 26% (n = 79) as T variants, however a significant proportion of the isolates (22%) represented either the intermediate (I) phenotype (n = 49) or mixtures of the different phenotypes (n = 18).

**Figure 2:**
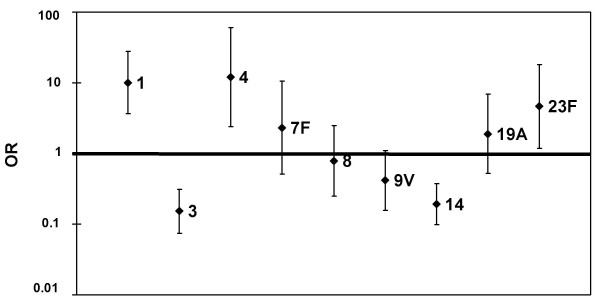
Serotype specific empiric odds ratio (OR) and 95% confidence intervals.

The prevalence of the different phenotypes among the serotypes was markedly different so we tested if there was an association between the O phenotype and particular serotypes. The results are summarized in figure 2. Five serotypes were found to have OR significantly different from unity, indicating an association with particular opacity phenotypes. Serotypes 1 (OR = 10.1, CI95% = 3.6–27.9), 4 (OR = 12.0, CI95% = 2.4–60.2) and serotype 23F (OR = 4.6, CI95% = 1.2–18.2) were more frequently associated with the opaque phenotype than expected whereas serotypes 3 (OR = 0.2, CI95% = 0.1–0.3) and 14 (OR = 0.2, CI95% = 0.1–0.4) were found associated with the transparent phenotype more frequently than expected. No serotype 12B isolates presented the T phenotype not allowing the determination of an OR value. We also tested if the O phenotype could be related to particular genetic lineages but failed to show any correlation possibly due to the smaller numbers (data not shown).

## Discussion

The analysis of a large collection (n = 304) of genetically diverse invasive isolates expressing the 10 most frequently found serotypes expanded on prior studies and confirmed the prediction from animal models and in vitro studies that the O phenotype should predominate among invasive isolates, supporting a role for pneumococcal phase variation in infections of human immunocompetent hosts. Notwithstanding, the T phenotype, with its lower capsule production and inferred higher sensitivity to opsonophagocytosis, was still expressed by a significant fraction of the isolates as was the I phenotype. Since its description together with the O and T phenotypes the I phenotype has remained poorly characterized [1]. Stability experiments conducted in our laboratory showed that reversion of the I phenotype to either the O or T phenotypes could be observed and occurred in the same frequency range (10^-3^-10^-6^) as spontaneous reversion between the O and T phenotypes [1]. In rare instances the reversion experiments resulted in a mixture where a high number of T or O colonies was found together with the I phenotype (data not shown). It is also interesting to note that serotypes 8 an 14 that presented high numbers of the I phenotype also showed the highest numbers of isolates presenting mixtures of the various phenotypes. We favor the interpretation that the I phenotype reflects a higher instability of some O phase variants and may be due to a lower capsule polysaccharide production in the absence of selective pressure in vitro. This is supported by the frequent observation that primary plates from normally sterile biological products yield more mucoid colonies than those observed after subculture in the laboratory and the fact that capsule production is strongly affected by changes in oxygen availability in O variants but not in T variants [8]. Further studies are needed to clarify the relationship between the I phenotype and the T and O phenotypes.

In spite of an overall higher frequency of the O phenotype in relation to the T phenotype (a 2:1 ratio, see table 1) a previously undescribed association between serotype and colony phenotype was noted. In evaluating the association between serotype and colony morphology we excluded all isolates presenting mixtures of the various phenotypes or the I phenotype due to the uncertainty of its relatedness to either the O or the T phenotype discussed above. Serotypes 7F, 8, 9V and 19A did not show a significantly higher association with the O phenotype whereas serotypes 1, 4 and 23F are more frequently O than expected by chance and none of the serotype 12B isolates presented the T phenotype. Both serotypes 1 and 4 are rarely isolated from healthy carriers and were associated with a higher invasive disease potential [9,10]. Moreover, serotype 1 is historically associated with outbreaks of serious pneumococcal disease, including a recent increase of pneumococcal meningitis in Ghana [11]. In the previous study of colonial opacity of isolates associated with human disease these two serotypes were also the most frequently recovered (n = 13/19) and in agreement with the data presented in this report, the isolates from blood presented mostly the O phenotype (n = 8/13) [8]. Although other bacterial factors will certainly affect the invasive disease potential, a predisposition of these serotypes for the O phenotype may help explain the epidemiology of their association with the human host [12]. Serotype 23F was also notable for a higher proportion of strains with the O phenotype but the study by Brueggmann et al. [10] found that this serotype was less associated with invasive disease than expected. However, that study also showed that individual clones expressing the same serotype have different abilities to cause invasive disease, suggesting that clone-specific virulence determinants might be important as well. In this respect it is notable that the genetic lineages identified in the study of Brueggmann et al. were different from the ones in this collection [10,13] raising the possibility that other clone related properties may have influenced the results of each study.

On the other hand, serotypes 3 and 14 were significantly less associated to the O phenotype than expected by chance. Serotype 14 is frequently carried and was also associated with a higher invasive disease potential, whereas serotype 3 was more frequently associated with carriage but it did not reach significance [9,10]. Although serotypes 3 and 14 were among the most frequent in our collection, together accounting for 35% of the isolates analyzed, the data suggest that the ability of these serotypes to cause human infections must be attributed to properties other than a preference for the O phase variant.

The usefulness of serotype as a marker for important biological characteristics of the pneumococcus has been recently confirmed [9,10,12]. Whether the serotype itself is the primary determinant of such characteristics or only a marker for a genetic background remains to be determined but numerous studies have also established the importance of certain clones in various aspects of pneumococcal disease raising the possibility of heterogeneity within serotypes [14]. Even if the smaller numbers of isolates of each clone represented in our sample prevented an unequivocal association of particular clones to each phenotype, we cannot exclude that such an association exists. However, we consider this unlikely due to the multiple genetic lineages, represented by various sequence types, found in each serotype (table 1).

The present study characterized only isolates responsible for invasive infections and future studies of isolates recovered from asymptomatic carriers may offer further insights into the role of phase variation in the biology of pneumococci. The recent finding that opaque variants may be required to establish long-term colonization, raises the possibility that carriage isolates may be less homogenous in terms of opacity phenotype than previously suggested [4]. Furthermore, the association of serotype and opacity phenotype described here suggests that other factors, in addition to the site of isolation of the strain, determine its opacity phenotype. Taken together these observations suggest a more complex distribution of opacity phenotypes than the clear association initially proposed between the opacity phenotype and the site of isolation.

It is known that the frequency of phase switch varies greatly [1] and this could introduce a bias in our analysis since most samples will have at least five passages in the laboratory before being scored for the opacity phenotype. Nevertheless, we do not believe that this compromises our conclusions because even considering the highest switch frequency described (10^-3^) one would expect to randomly pick a colony of the minority phenotype only 0.1% of the times (a cumulative error of 0.5% after five passages). It is important to note however, that this estimate assumes that no selection occurs for any of the phenotypes in in vitro growth and passage.

## Conclusion

We found that the O phenotype predominated on a 2:1 ratio over T variants among a large collection (n = 304) of isolates representing diverse genetic backgrounds and 10 serotypes associated with human invasive disease, supporting a role for pneumococcal phase variation in human infections. However, the data also revealed an asymmetric distribution of O variants between serotypes. This heterogeneity of opacity phenotypes in human invasive isolates further supports the proposal that other factors, in addition to the site of isolation, determine the opacity phenotype of a given isolate. The newfound association of the O phenotype with certain serotypes could offer new insights into the reasons for the epidemiological differences observed among pneumococcal serotypes, in particular the potential of certain serotypes to cause invasive disease.

## Methods

### Bacterial strains and growth conditions

The collection of isolates analyzed was characterized previously [13,15]. The colony phenotype of a subset representing 36 different sequence types found among the 10 most prevalent serotypes (n = 304) was determined. Bacterial cultures stored at -70°C were plated onto tryptic soy agar (Difco, Becton Dickinson, Erembodegem, Belgium) plates supplemented with 5% sheep blood and incubated overnight at 37°C in a 5% CO_2 _atmosphere. For the scoring of the dominant phenotype cells were suspended in saline to McFarland 0.5 and diluted appropriately to obtain 10^4 ^cfu in 100 μl that were then plated in duplicate in tryptic soy agar plates onto which 5,000U of catalase (Sigma – Aldrich, Sintra, Portugal) was spread. We tested several conditions and found that the time of incubation and differences in the availability of oxygen affected colony appearence, in agreement with previous publications [8]. We found that the use of vented Petri dishes and incubation at 37°C in a candle extinction jar for 16 h provided the best conditions to distinguish the different phase variants.

### Colony morphology and scoring of the dominant phase phenotype

Colony morphology was determined on transparent medium using a stereomicroscope with a substage illuminator under oblique transmitted illumination as previously described [1]. We considered the three possible phenotypes described to date: opaque (O), transparent (T) and intermediate (I) [1]. In agreement with previous observations [8] most isolates presented colonies of various phenotypes on the plates, albeit in different number, facilitating the assignment of the dominant phenotype. An isolate was scored as expressing the O, T or I phenotype if >70% of the colonies visible in two plates were of a single phenotype. If none of the observed phenotypes accounted for >70% of the colonies the strain was classified as a mixture of multiple phenotypes. To facilitate a large scale phenotype assignment the plates were divided into sectors that were visually inspected for colony phenotypes. If >70% of the surface area of each sector was covered in colonies of a single phenotype the strain was classified accordingly. Since there was total agreement between the classification obtained by total colony counts and visual estimation of surface area coverage, the simpler method was adopted and any uncertainties were resolved by colony counts.

### Statistical analysis

In order to compare the probability of association of particular serotypes with the O phenotype, an empirical odds ratio (OR) and 95% confidence intervals (CI) [16] were calculated by reference to all other serotypes. An OR of 1 indicates that the serotype was equally likely to be opaque or transparent, whereas an OR>1 or OR<1 indicated an increased or reduced probability to be opaque, respectively. Only results whose 95% CI did not cross unity were considered significant.

The OR was calculated as follows: OR = (ad)/(bc), where a is the number of opaque A serotype or clone, b is the number of transparent A serotype or clone, c is the number of non-A serotypes or clones and d is the number of transparent non-A serotypes or clones. It follows from the presented formula that it is not possible to calculate an OR value when none of the isolates of a given serotype or clone presented the transparent phenotype.

The choice of using all other serotypes or clones to measure the reference OR was substantiated by prior studies (reference 10 and references therein) that also provide a discussion on the strong points of this method.

## Authors' contributions

IS carried out the colony observations, the stability experiments and drafted the manuscript. JMC participated in the design of the study and helped to draft the manuscript. MR conceived the study, and participated in its design and coordination, performed the statistical analysis and helped to draft the manuscript. All authors read and approved the final manuscript.

## Supplementary Material

Additional File 1 Colonies of invasive pneumococci expressing 10 different serotypes representing various phenotypes. Panels on the left represent mostly colonies with an opaque phenotype whereas panels on the right represent fields with mostly transparent colonies or mixtures of the various phenotypes. a) Serotype 1; left panel magnification ×20, right panel magnification ×25. b) Serotype 3; left ×12, right ×8. c) Serotype 4; right ×25, left ×25. d) Serotype 7F, left ×25, right ×32. e) Serotype 8, left ×20, right ×12. f) Serotype 9V, left ×16, right ×20). g) Serotype 12B, left ×20, left ×16. h) Serotype 14, left ×25, right ×25. i) Serotype 19A, left ×20, right ×12. j) Serotype 23F, left ×20, right ×12. Click here for file

## Figures and Tables

**Table 1:** Genetic diversity, serotypes and colonial opacity of invasive isolates (n = 304)

**Serotype**	**No. of STs ^a^**	**No. of isolates with phenotype**				**Total**
			
		**Opaque (O)**	**Transparent (T)**	**Intermediate (I)**	**Mixture**	
1	4	45	3	1	1	50
3	5	11	26	7	1	45
4	3	21	1	4	1	27
8	2	8	5	10	4	27
14	8	15	28	10	9	62
12B	4	13	0	2	0	15
19A	6	11	3	3	0	17
23F	3	17	2	2	1	22
7F	1	9	2	7	0	18
9V	5	8	9	3	1	21
**Total**	- ^b^	158	79	49	18	304

^a^Distinct sequence types (ST) with serotype as determined by multi-locus sequence typing [13].

^b^Since five STs were found associated with more than one serotype the number of unique STs is 36
