# Supplementary material for: Heterogeneity of pneumococcal phase variants in invasive human infections
Source: BMC Microbiol. 2006 Jul 26;6:67. doi: 10.1186/1471-2180-6-67 (PMC1555587; doi:10.1186/1471-2180-6-67)
Supplement: Additional File 1 — Colonies of invasive pneumococci expressing 10 different serotypes representing various phenotypes. Panels on the left represent mostly colonies with an opaque phenotype whereas panels on the right represent fields with mostly transparent colonies or mixtures of the various phenotypes. a) Serotype 1; left panel magnification ×20, right panel magnification ×25. b) Serotype 3; left ×12, right ×8. c) Serotype 4; right ×25, left ×25. d) Serotype 7F, left ×25, right ×32. e) Serotype 8, left ×20, right ×12. f) Serotype 9V, left ×16, right ×20). g) Serotype 12B, left ×20, left ×16. h) Serotype 14, left ×25, right ×25. i) Serotype 19A, left ×20, right ×12. j) Serotype 23F, left ×20, right ×12. [file 1471-2180-6-67-S1.pdf]

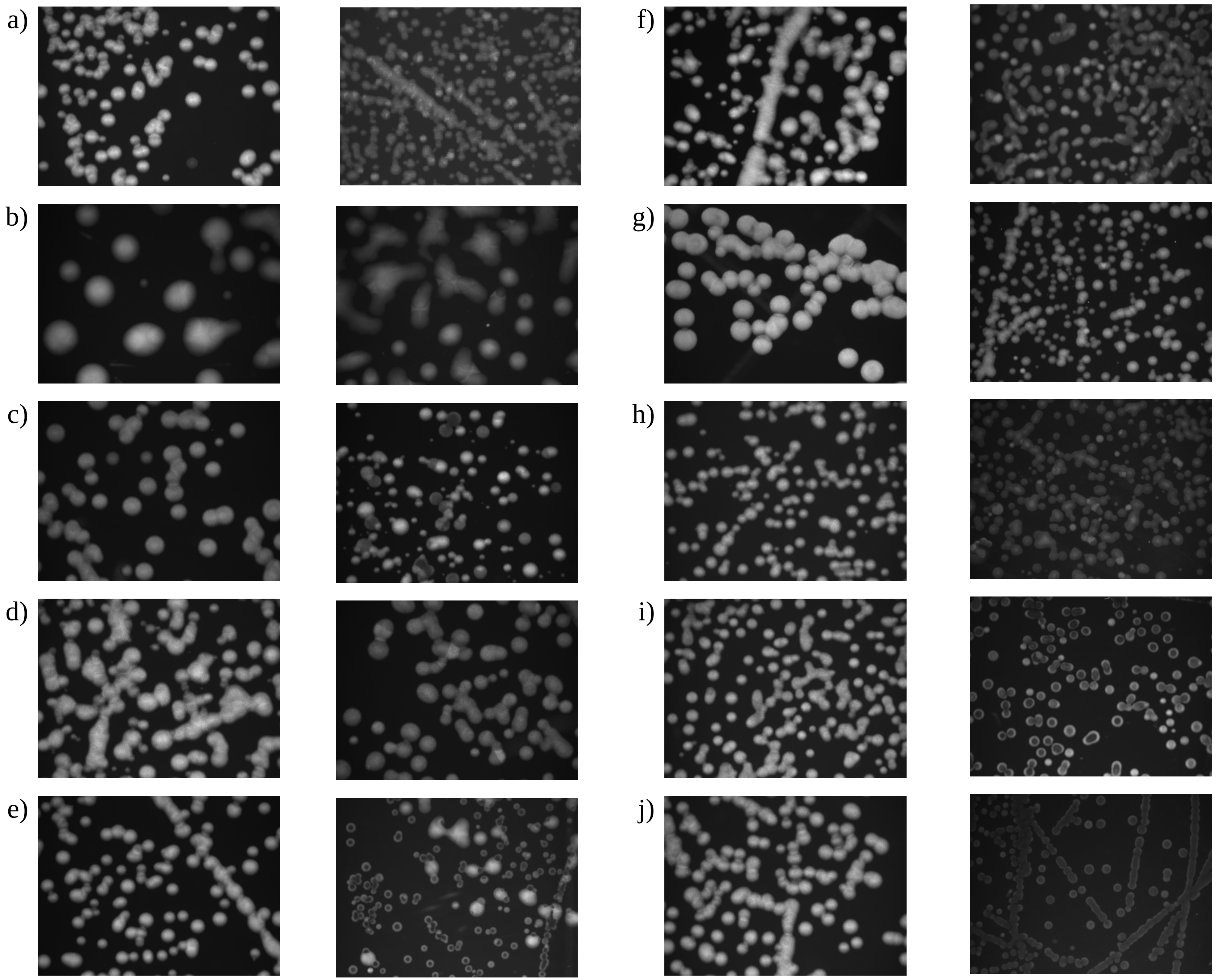
a)
b)
c)
d)
e)
f)
g)
h)
i)
j)

**Supplemental figure**

**Colonies of invasive pneumococci expressing 10 different serotypes representing various phenotypes.** Panels on the left represent mostly colonies with an opaque phenotype whereas panels on the right represent fields with mostly transparent colonies or mixtures of the various phenotypes. a) Serotype 1; left panel magnification ×20, right panel magnification ×25. b) Serotype 3; left ×12, right ×8. c) Serotype 4; right ×25, left ×25. d) Serotype 7F, left ×25, right ×32. e) Serotype 8, left ×20, right ×12. f) Serotype 9V, left ×16, right ×20). g) Serotype 12B, left ×20, left ×16. h) Serotype 14, left ×25, right ×25. i) Serotype 19A, left ×20, right ×12. j) Serotype 23F, left ×20, right ×12.
